# Void Avoidance Opportunistic Routing Protocol for Underwater Wireless Sensor Networks

**DOI:** 10.3390/s21061942

**Published:** 2021-03-10

**Authors:** Rogaia Mhemed, Frank Comeau, William Phillips, Nauman Aslam

**Affiliations:** 1 Department of Engineering Mathematics and Internetworking, Dalhousie University, Halifax, NS B3H 4R2, Canada; Rogaia.Mhemed@dal.ca (R.M.); William.phillips@dal.ca (W.P.); 2 Department of Engineering, St. Francis Xavier University, Antigonish, NS B2G 2W5, Canada; 3 Department Computer and Information Sciences, Northumbria University, Newcastle upon Tyne NE1 8ST, UK; nauman.aslam@northumbria.ac.uk

**Keywords:** Underwater Sensor Networks (UWSNs), Opportunistic Routing (OR), void node, hop count, energy consumption, Packet Delivery Ratio (PDR)

## Abstract

Much attention has been focused lately on the Opportunistic Routing technique (OR) that can overcome the restrictions of the harsh underwater environment and the unique structures of the Underwater Sensor Networks (UWSNs). OR enhances the performance of the UWSNs in both packet delivery ratio and energy saving. In our work; we propose a new routing protocol; called Energy Efficient Depth-based Opportunistic Routing with Void Avoidance for UWSNs (EEDOR-VA), to address the void area problem. EEDOR-VA is a reactive OR protocol that uses a hop count discovery procedure to update the hop count of the intermediate nodes between the source and the destination to form forwarding sets. EEDOR-VA forwarding sets can be selected with less or greater depth than the packet holder (i.e., source or intermediate node). It efficiently prevents all void/trapped nodes from being part of the forwarding sets and data transmission procedure; thereby saving network resources and delivering data packets at the lowest possible cost. The results of our extensive simulation study indicate that the EEDOR-VA protocol outperforms other protocols in terms of packet delivery ratio and energy consumption.

## 1. Introduction

Water covers more than two thirds of the earth’s surface. This environment is very important for human life since it plays an important role as a transportation medium and affects the earth’s climate as well as global production because of its richness in natural resources. Due to these reasons, lately, researchers have been giving more attention to Underwater Sensor Networks (UWSNs) to investigate and discover the unexplored submerged underwater areas and empower numerous applications such as resource exploration, oceanographic data collection, pollution monitoring, tactical surveillance, oil/gas spills monitoring, etc. [1,2,3,4,5,6,7]. To make such applications realistic, effective communication protocols are fundamentally required to complete the communication procedure between the underwater devices successfully. Many communication protocols have been proposed to address different Terrestrial Wireless Sensor Networks (TWSNs) issues [8,9], which make such networks widely investigated. On the other hand, many UWSN issues remain open and need more investigation.

UWSNs have different characteristics and features when compared to TWSNs [10]. These differences can be seen in many aspects. First, UWSNs use acoustic signals for communication instead of radio signals used by TWSNs. Second, the UWSNs’ topologies are more dynamic than the topologies of TWSNs. Third, the underwater deployment is unattended and comparatively sparse as compared to TWSNs. Fourth, node localization in UWSNs is hard compared to node localization in TWSNs. Fifth, underwater sensor nodes have more costly hardware than terrestrial ones, and they are resource limited (i.e., memory and energy). Moreover, it is hard to replace or recharge their batteries after deployment.

As we mentioned above, UWSNs use acoustic signals while TWSNs normally use radio signals. While the radio signals do not propagate well and suffer from attenuation in underwater environments, the acoustic signals perform wireless communication in underwater environments with satisfactory range, a smaller amount of attenuation and higher reliability [11]. The acoustic signal is affected by underwater characteristics such as strong attenuation and ambient noise, time-varying multipath propagation, and low-speed sound propagation (speed of sound in water is ≈1500 m/s, which is five orders of magnitude lower than the radio signals used in TWSNs) [7,12]. These underwater properties result in high delay and error rate, temporary loss of connectivity, limited bandwidth capacity, and high-energy communication cost.

Since UWSNs use acoustic signals in their communication, the straightforward application of the traditional TWSN protocols to UWSNs reduces network performance [2,3,11]. Consequently, several underwater protocols were proposed to improve communication in underwater networks. These proposed protocols for UWSNs have considered various underwater parameters and addressed different problems. Improving network lifetime is a significant objective in UWSNs since replacing or recharging batteries of underwater nodes is a very expensive and difficult task in the harsh underwater environment. Two or more nodes may become unreachable as node battery energy depletes, causing a topology partition that results in the void area problem. This issue has attracted the attention of many researchers. 

In addition to the void zone problem, acoustic signal fading degrades routing protocol performance. Opportunistic Routing (OR) has been proposed as a novel technique to improve network function by mitigating high bit errors and losses due to shadow zones, limited bandwidth, high power consumption, and signal spreading [6].

It is known that in wireless networks the energy expended by sensor nodes in transmitting a data packet is more than the energy expended in receiving it. Hence, to improve the network lifetime, save the network resources and keep network connectivity, the rate of node energy consumption should be decreased by reducing the number of transmissions and therefore reducing the number of packets that must be forwarded from a source towards a sink(s).

In summary, harsh underwater environment characteristics, underwater sensor nodes’ limited resources and acoustic signal limitations increase the probability of bit errors, packet loss and network partition, decreasing network performance. The possibility of addressing these challenges using promising OR features motivated us to develop a void avoidance routing protocol for UWSNs. This protocol increases network performance by excluding all the routes that would lead to the loss of data packets.

In this paper, we propose a novel reactive routing protocol for UWSNs that addresses the void area problem. Energy Efficient Depth-based Opportunistic Routing with Void Avoidance for UWSNs (EEDOR-VA) utilizes the node’s hop count from a sink to select a next-hop forwarder set that can continue forwarding the packet towards the sink(s). In EEDOR-VA, the low priority nodes suppress their transmissions whenever they sense the same packet was sent by a high priority node to avoid redundant transmissions and their related costs. The proposed protocol’s novelty lies in a hop count discovery mechanism inspired by the Dynamic Source Routing (DSR) algorithm proposed in [13] to update a node’s hop count from the sink and integrating hop count discovery with a novel OR technique that comprises two building blocks—candidate forwarding set selection and candidate set coordination [14]. Instead of the periodic messages from the sinks implemented by most of the routing protocols, we propose a hop count discovery process that consists of Hop Count Request (HCREQ) and Hop Count Reply (HCREP) procedures to find the hop count of the sensor nodes. The idea is to remove void/trapped nodes that are in the current packet holder node’s neighborhood from that node’s forwarding set. The simulation results of low and high density scenarios showed that EEDOR-VA is able to decrease the total energy consumption, reduce the number of transmitting nodes and increase the packet delivery ratio.

This work enhances our previous routing protocol [15] by designing an opportunistic routing protocol to cope with underwater acoustic channel weaknesses and address the void area problem in underwater network scenarios. 

Our new protocol is better than existing underwater routing protocols mainly by implementing the hop count discovery, which reduces the network overhead caused by periodic beacons and retransmissions and improves the packet delivery ratio while also increasing energy efficiency, since packets are only transmitted if a path is found from the source to the sink(s).

More discussion on the EEDOR-VA contributions is listed below:

- Implementing the proposed hop count discovery technique inspired by DSR instead of flooding periodic beacons widely used in the literature ensures less network communication overhead and lower network resource consumption.
- The small size of proposed HCREQ and HCREP messages that are used to discover the hop count between the packet generator and the sink(s) reduces the collisions and network overhead and decreases the total energy consumption.
- Forwarding set coordination based on the proposed waiting time supports balancing energy consumption by suppressing retransmissions.
- The development of the forwarding set selection procedure excludes all void/trapped nodes that may lead to a void area from the data routing path. Our proposed protocol checks the node reachability to the sink and updates in-route node hop counts. If a node does not have any path that facilitates forwarding the packet toward the sink(s), the node is dropped from the forwarding candidate set.
- Multiple opportunistic routing paths established through hop count discovery increase the packet delivery ratio.

Our novel protocol is a reactive loop-free OR protocol proposed to enhance EEDOR by addressing the void area problem based on the hop count discovery of the source node. This approach improves the packet delivery ratio by avoiding paths that have a large number of hops that increase the packet error rate as a result of packet collisions.

The rest of this paper is organized as follows. In Section 2, some of the well-known related works in the area of void avoidance are reviewed. The details of our protocol are presented in Section 3. The simulation results of different scenarios are presented in Section 4. Finally, the conclusion of our study is provided in Section 5.

## 2. Related Works

A number of protocols have been proposed that use OR techniques to deal with the void communication area problem. In this section, we first give a brief classification of routing protocols found in the literature that address the void problem as well as other comparative protocols. We then present a review and discussion of these protocols and their benefits and drawbacks. 

**Figure 1:**
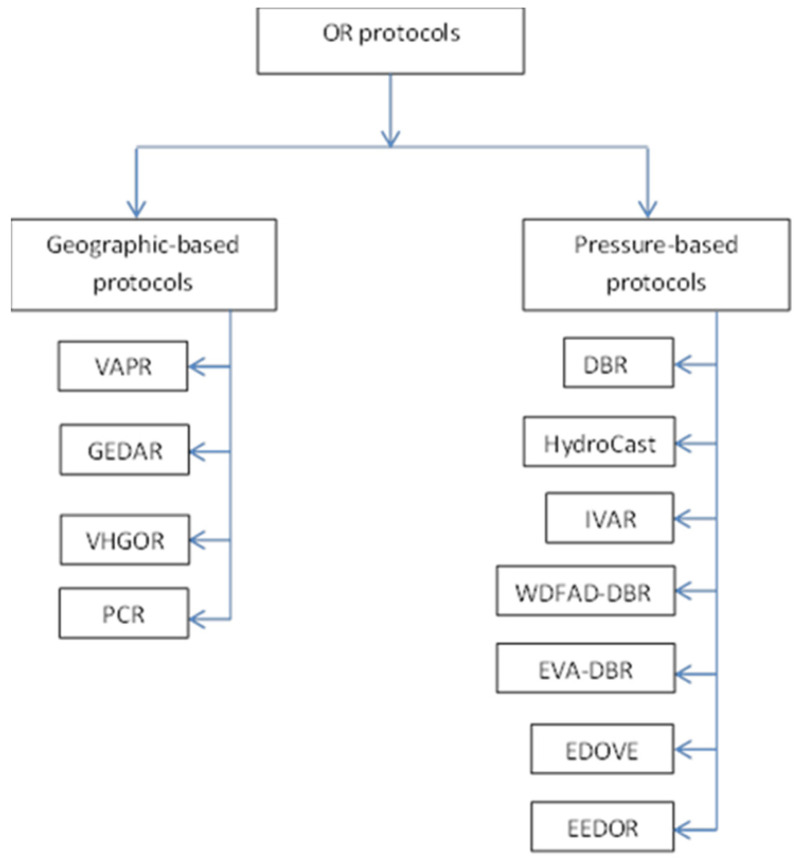
Classification of Opportunistic Routing (OR) protocols based on positioning information. Void-Aware Pressure Routing (VAPR), Geographic and opportunistic routing with Depth Adjustment-based topology control for communication Recovery (GEDAR), Void handling using Geo-Opportunistic Routing in underwater wireless sensor networks (VHGOR), a novel power controlled opportunistic routing protocol for internet of underwater things (PCR), Depth-Based Routing (DBR), Pressure Routing for Underwater Sensor Networks (HydroCast), Inherently Void Avoidance Routing Protocol for Underwater Sensor Networks (IVAR), weighting depth and forwarding area division DBR routing protocol (WDFAD-DBR), Energy-efficient and Void Avoidance Depth Based Routing (EVA-DBR), Energy and Depth variance-based Opportunistic Void avoidance (EDOVE), Energy Efficient Depth-Based Opportunistic Routing protocol (EEDOR).

The existing OR protocols in UWSNs can be classified into two main categories based on their positioning information: Geography-based routing protocols and Pressure-based routing protocols. This classification can be seen in Figure 1.

In the Geography-based category such as [16,17,18,19], selecting the forwarding set candidates and making the forwarding packet decisions in OR requires information about the geographic location of sensor nodes, while in the Pressure-based category, as in [15,20,21,22,23,24,25], the depth information of nodes is needed to select the next forwarding set candidates and make forwarding packet decisions.

Void-Aware Pressure Routing (VAPR) [16] is an anycast soft-state routing protocol that was proposed to address the void node issue in UWSNs. VAPR consists of two stages: an enhanced beaconing stage and an opportunistic data forwarding stage. Instead of encountering a void area and then implementing a recovery mode, VAPR takes advantage of geographic routing and employs the regular beaconing messages method, which includes some useful local information about the node to be used in the forwarding set selection stage. VAPR suffers from high-energy consumption since it measures the distance to the neighboring nodes and uses enhanced beaconing to exchange node information between the neighbors periodically.

In [17], Geographic and opportunistic routing with Depth Adjustment-based topology control for communication Recovery (GEDAR) utilizes the greedy forwarding technique by knowing the position information of each current forwarding node, its neighbors, and the known sink. GEDAR is a sender-side OR technique, where the forwarding set of candidates is determined in each hop by the sender node. GEDAR uses network topology control techniques to increase the connectivity of the network and reduce the number of packet retransmissions. Moreover, the topology control method is also utilized to address the void zone problem. On the other hand, the depth adjustment technique used by void nodes to move to a new depth that allows them to communicate with other node(s) consumes a significant amount of energy. Another drawback of GEDAR is that sensor node energy is not considered when forwarder nodes are selected, which may result in poor forwarder node selection.

Void handling using Geo-Opportunistic Routing in underwater wireless sensor networks (VHGOR) [18] adopts Geography-based Opportunistic Routing (GOR) to forward data packets to reach the destination over multi-hops. It is a heuristic protocol implemented with two methods to form optimal forwarder selection. The first metric is Opportunistic Routing based Expected Packet Progress (OREPP), which is calculated based on the difference between the geographic distance between the source and destination, and the geographic distance between any node and the destination, residual energy and packet delivery probability. The second metric is Node Closer to the Destination (NCD); NCD can be defined as the best node with maximum OREPP to forward the current packet. VHGOR uses a greedy forwarding approach to advance the packet towards the destination, and if a packet gets into a void node, the protocol switches to the void mode. The limitations of VHGOR include consuming restricted resources such as node memory, through maintaining a neighboring table, and the node energy through node beacons.

Recently, a novel power control-based opportunistic (PCR) routing protocol for internet of underwater things (IoUTs) was proposed in [19]. To achieve energy-efficient data delivery in IoUTs, the authors designed an opportunistic routing protocol that includes transmission power control. In PCR, each node considers more than one transmission power level to choose its candidate set for each next-hop, and then the energy waste for each candidate set is calculated to determine the appropriate transmission power level and the next-hop forwarding set. The packet delivery ratio is increased by modifying the transmission power level at each hop in order to choose the appropriate candidate node set from the sender neighbors to continue forwarding the data packets to reach the sink(s) on the water surface. PCR also reduces the transmission power level when the number of deployed nodes is increased to reduce the number of retransmissions, which decreases the energy consumption in some cases. However, as we can see from the results presented, the energy consumption is greater than that in the compared related works, which will affect the network lifetime.

The Depth-Based Routing (DBR) presented in [20] was the first OR protocol proposed for UWSNs using sensor node depth. In DBR, the current forwarding node uses the flooding technique to send the packet to its neighbors. The receiving neighbors then decide to be a forwarding candidate by comparing their depth with the depth in the received packet. The depth threshold is also implemented during the forwarding set formation to select the nodes that are far from the current forwarder to continue the forwarding process. The holding time used to manage the coordination phase between the forwarding nodes is determined based on node depth. Using only the depth of the sensor nodes as a metric for forwarding set selection reduces the protocol’s performance because the nodes with smaller depths are involved in the forwarding process most of the time. Therefore, such nodes drain their energy before the other nodes in the network, which generates void zones. Moreover, the DBR flooding technique increases the probability of packet delivery to the surface but also increases redundant packets and packet retransmissions. Consequently, an extreme amount of energy will be consumed. In DBR, redundant packets and packet retransmissions happen because of the node’s holding time. That is, nodes may have the same depth, and using only the depth in calculating the hold time will give a number of nodes the same transmission times.

In [21], the Pressure Routing for Underwater Sensor Networks (HydroCast) protocol is presented. HydroCast applies only the local information of the topology to form a cluster with nodes excluding hidden-terminal among them, and at the same time maximizing the Expected Packet Advance (EPA) of this cluster. In HydroCast, the current forwarder node needs to know the two-hop connectivity and the pairwise distances for the neighboring nodes. Nodes in the forwarding set are prioritized using a distance-based timer approach that results in the most distant node from the source having the shortest timer, and so on, to help in scheduling the transmissions and suppressing collisions. HydroCast addresses the void area issue using an OR approach, which also enhances the packet delivery ratio with small end-to-end delays since a subset of the neighboring nodes simultaneously receive the data packet properly. However, as a result of using OR, HydroCast suffers from redundant packet transmissions where a data packet may be delivered to the sink multiple times, causing the depletion of network resources. In addition, implementing the recovery mode increases the energy cost, and the simulation results presented in the paper do not show any details about the energy consumed by the pressure sensor in order to find its depth.

Inherently Void Avoidance Routing Protocol for Underwater Sensor Networks (IVAR) [22] is a receiver-based forwarding protocol where the forwarding node does not need to store its neighbor’s information. In IVAR, a hop-by-hop forwarding set selection technique is used to forward the data packets from the sensed node to the sink. Each packet holder uses local information of hop distance and packet advancement to determine its own forwarding set. The nodes in these forwarding sets are given a priority depending on two metrics to forward packets: their hop count as a first metric and their depths as a second one. IVAR suffers from redundant packet transmission due to a hidden node problem. Consequently, redundant packet transmissions lead to an increase in energy consumption.

In [23], another pressure-based routing protocol is described, namely weighting depth and forwarding area division DBR routing protocol (WDFAD-DBR). To increase the reliability of the packet transmission and decrease the probability of the void area problem, WDFAD-DBR uses the weighting depth difference of two-hop nodes to construct its routing decision. Broadcast control packets and acknowledgment messages (ACKs) periodically consume the node’s battery and memory. Duplicated packets are handled by dividing the forwarding area and using a neighbor node prediction mechanism that helps to reduce the energy consumption. Void holes are avoided by using the depth of the expected next hop. However, retransmission is required if the best forwarding node fails to transmit the packet. Additionally, routing flexibility might be affected due to choosing a fixed primary forwarding area to form the forwarding set so that a void area is not dealt with completely since trapped nodes are not eliminated from the forwarding set.

The Energy-efficient and Void Avoidance Depth Based Routing (EVA-DBR) protocol was proposed in [24]. The EVA-DBR routing protocol consists of two phases: an updating phase and a routing phase. The protocol depends on the information broadcasted periodically in the updating phase from the neighbor nodes that are one-hop away from the source node for void detection and bypassing in the routing phase. Using a forward area resizing technique helps in addressing the hidden node problem and, in some cases, can also detect the void and trapped nodes before the data packet gets caught in a void node. However, in both phases of the protocol, periodically broadcasting neighbor information consumes node resources and many duplicate packet transmissions seem to happen in sparse networks. In addition, the hidden node problem may be present if the forwarding range is chosen to be more than half of the transmission range.

In [25], a protocol called the Energy and Depth variance-based Opportunistic Void avoidance (EDOVE) protocol was presented based on WDFAD-DBR, the work presented in [23]. The protocol handles the void area problem by choosing the forwarder candidates among the total distributed nodes that have a large residual energy and have several nodes in their transmission range (neighbors). To obtain this useful node information, each node in the network topology exchanges its information with its one-hop neighbors through the neighbor request and neighbor acknowledgment packets, and each node has to maintain its neighbor table. EDOVE considers energy level as one of its parameters to help in reducing energy consumption and avoiding energy holes. However, exchanging neighbor information and maintaining the neighbor table consumes node resources. The protocol also suffers from duplicate packet transmissions, which increases energy consumption. Moreover, the void area is not handled completely since the protocol only addresses energy void holes.

Most recently, an Energy Efficient Depth-Based Opportunistic Routing protocol (EEDOR) was presented in [15]. EEDOR is a hybrid forward set selection procedure where the forwarding candidate set is selected in a cooperative way between the current forwarder node and its neighbors. In EEDOR, the current forwarder node’s forwarding set contains only the candidate neighbors with less depth than the current forwarder. Moreover, the nodes in the forwarding candidate sets are given priorities based on their depths. These priorities are used in the novel holding time proposed by the protocol to determine the transmission time for each node in the forwarding set candidates. The proposed holding time reduces the collisions and retransmissions issue caused by multiple nodes having equal transmission times. The protocol enhances the lifetime of UWSNs, decreases the total energy consumption and reduces the number of nodes involved in the packet forwarding procedure. In contrast, EEDOR suffers from a low packet delivery ratio in the simulated network topology. In addition, the protocol does not address the void area problem.

Advantages and disadvantages of the OR protocols for UWSNs related to our work discussed above are summarized in Table 1.

## 3. EEDOR Void Avoidance with Hop Count Discovery Mechanism 

### 3.1. Network System 

Our UWSN architecture model consists of a number of sensor nodes randomly deployed underwater in different depths and multiple immobile sinks located on the water’s surface. The sinks are equipped with both radio modems to communicate with each other and/or with a base station, and acoustic modems to communicate with the underwater sensor nodes. The underwater sensor nodes consist of two types: nodes that participate in packet forwarding and nodes that do not. Nodes that participate in packet forwarding are the source node, next forwarder nodes, and forwarding candidates. Nodes that do not participate in packet forwarding are void nodes, trapped nodes, and idle nodes. Each type of node is defined as follows:

1. Nodes that participate in packet forwarding.
  - The source node is a node that senses the phenomena and has the collected data to send to the sink(s) on the water’s surface.
  - Next forwarder nodes are the source nodes for the next hops that will continue the forwarding procedure.
  - Forwarding candidates are other candidate nodes in the forwarding set that may become sources for the next hop if the higher priority candidates fail in forwarding the data packets.
2. Nodes that do not participate in packet forwarding.
  - Void nodes are nodes that do not have any neighbor nodes with less depth than themselves and are not within range of a sink. They therefore cannot send data to any node that could help in the forwarding process and deliver the packet to its destination.
  - Trapped nodes are nodes in which the only node in transmission range with less depth than itself is a void node or a node whose only path to nodes of lesser depth leads to a void node. That is, the only nodes of lesser depth that are in range of a trapped node are a void node or other trapped nodes.
  - The idle nodes are those nodes that are not part of a given source to sink transmission process.

**Figure 2:**
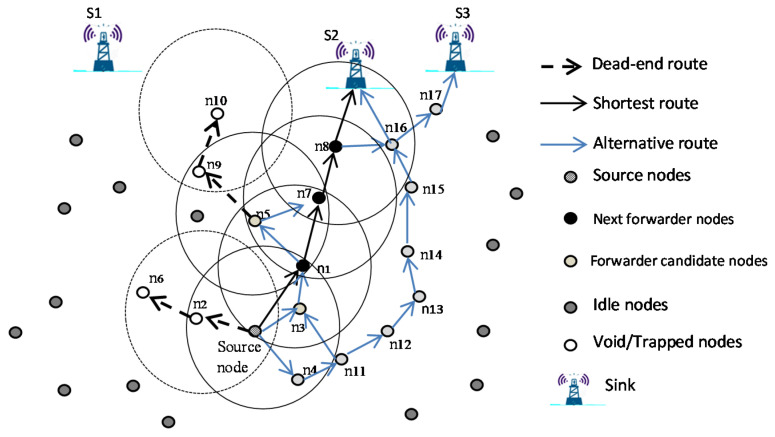
Network Architecture.

In our proposal, we improve the routing performance of EEDOR presented in [15] by using on-demand hop count discovery to prevent the void and trapped nodes from participating in the data packet transmission, to save the network resources. The network scenario shown in Figure 2 illustrates our model and the routing paths. 

In this model, nodes are homogeneous in terms of energy consumption and transmission range. When the packet holder (P_holder_) node has a packet to send to the surface sinks, it will use the neighboring relay nodes determined by the hop count discovery process to deliver this packet. The P_holder_ and its neighboring relay nodes use acoustic signals to transmit their packets. We used the Thorp propagation model described in the next section to model the underwater acoustic channel.

### 3.2. Acoustic Channel Model

Sensor nodes in wireless sensor networks are battery-powered devices. Nodes in UWSNs consume a significant amount of energy because of the underwater acoustic channel characteristics. When the sensor nodes deplete their batteries, the sensor networks eventually cannot operate correctly. In this section, we describe the path loss and ambient noise of the underwater acoustic channel as in [26,27]. 

The sonar equation, which characterizes the signal-to-noise ratio (*SNR*) of passive sonar, is shown in Equation (1) below, as presented in [26,28,29,30]: 

The model starts with the passive sonar equation, which describes the *SNR* in dB:

*SNR* = *SL* − *TL* − *NL* + *DI* (1)

where *SL* is source level, *TL* is transmission loss, *NL* is noise level and *DI* is directivity index. *DI* = 0 as denoted in [29].

**Source Level (*SL*):** Referring to [30], we can calculate *SL* as:

*SL* = 170.8 + 10 × log(*P_tx_*) (2)

where *P_tx_* is the total acoustic power of the source.

**Transmission loss (*TL*):** Referring to [26,31] we can calculate the *TL* as the sum of both spreading and absorption loss, as follows in Equation (3)

*TL* = *k* × 10 log(*d*) + *αd* × 10^−3^ (3)

where *d* is the distance in *km*, *α* is the absorption coefficient, expressed in Equation (4), and *k* is the spreading factor (*k* = 1 is cylindrical, *k* = 2 is spherical, and *k* = 1.5 in practical spreading). 

**Absorption loss (*α*):** We use Thorp’s expression since it is widely used in the publications [26,29,30]. According to [26], the absorption coefficient (*α*), which depends on frequency (*f*), can be defined and expressed as in Equation (4)

*α* = 0.11 × *f*^2^/(1 + *f*^2^) + 44 × *f*^2^/(4100 + *f*) + 2.75 × 10^−4^ + 0.003 (4)

where *f* is the frequency in kHz.

**Noise Level (*NL*):** The noise levels in the ocean have a serious influence on the acoustic channel; they can be divided into [30,31,32]:

- Ambient Noise: This noise is due to seismic and biological phenomena and water movement, which includes tides, current, storms, wind, and rain.
- Man-made noise: This is unnatural noise caused by human and shipping activity such as pumps, reduction gears and power plants, especially in areas encumbered with heavy vessel traffic.

Four sources of noises are used to model the noise level, namely: turbulence (*N_t_*(*f*)), shipping (*N_s_*(*f*)), wind (*N_w_*(*f*)), and thermal (*N_th_*(*f*)) noises. Equation (5) shows the calculation of these four factors in dB/Hz.

(5) 10×logNt(f)=1−30×log(f)10×logNs(f)=40+20×(s−0.5)+26×log(f)−60×log(f+0.03)10×logNw(f)=50+7.5×(w)12+20×log(f)−40×log(f+0.4)10×logNth(f)=−15+20×log(f)

where *s* defines a shipping activity factor value ranging from 0 to 1, *w* gives the wind speed in m/s and *f* is the frequency in kHz.

Then, the overall noise is expressed in Equation (6)

(6) $$NL=10\times \log\left((N_{t}\left(f\right)+N_{s}\left(f\right)+N_{w}\left(f\right)+N_{th}\left(f\right)\right)\times B)$$

where *B* is the receiver bandwidth in Hz.

**Bit Error Rate (BER):** We assume that the underwater acoustic micro-modem uses binary phase shift keying (BPSK) modulation [17,19,33]. Accordingly, the bit error rate (*P_e_*) of BPSK in an underwater Rayleigh fading channel can be calculated as in [33]:

(7) Pe=12×(1−10SNR101+10SNR10)

where *SNR* is the signal-to-noise ratio defined by Equation (1).

**Probability of Successful Packet Delivery:** For a packet of *m* bits, the probability of successful packet delivery (*P*) is:

(8) $$P={\left(1-P_{e}\right)}^{m}$$

**Figure 3:**
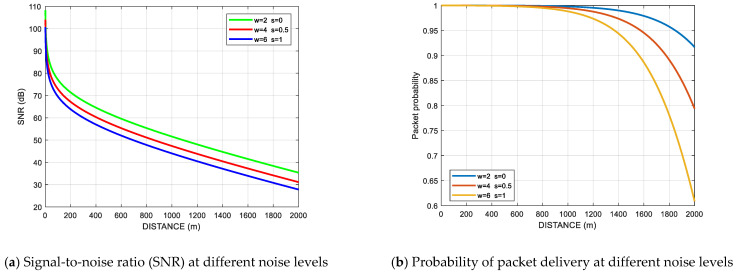
Impact of noise level on (**a**) *SNR*, (**b**) probability of packet delivery.

Figure 3a illustrates the impact of the channel noise level and the transmission loss on *SNR* for various values of wind and shipping noise. The impact of these effects on packet delivery probability is shown in Figure 3b.

### 3.3. Description of the Proposed EEDOR-VA Protocol

In this paper, we extend our previous EEDOR protocol and enhance the network performance by addressing the void area problem. 

Our technique to address the void area problem consists of bypassing the void and trapped nodes. EEDOR-VA makes a routing decision according to the nodes’ reachability to the surface sink(s). Our novel hop count discovery process was inspired by the source route discovery process used in DSR. In EEDOR-VA, Hop Count Request (HCREQ) and Hop Count Reply (HCREP) messages are used to update the node’s hop count to the reachable closest sink. The void and trapped nodes in the transmission range of a source and/or relay nodes do not reply to the HCREQ message and are excluded from being one of the forwarding candidates, so the data packet is not trapped in these nodes. Therefore, each P_holder_ can form its forwarding set easily. Since the presence of the void area can prevent communication between some of the network nodes, which reduces the routing protocols performance, our goal is to increase the routing performance through developing a routing protocol that gains a high packet delivery ratio with less energy consumption by selecting the shortest routing path. To complete a successful transmission, each packet must reach the next forwarder successfully at each hop and one of the sinks at the end of the forwarding procedure. The information received from the hop count discovery algorithm is used to assist the sensor nodes to update their hop count from the sink(s) and exclude the void and trapped nodes in the P_holder_ nodes’ transmission range. The hop count discovery mechanism in EEDOR-VA eliminates periodic beaconing and its associated costs.

The main idea of EEDOR-VA is to determine multiple loop-free paths between a source node and a single sink or multiple sinks on the sea surface. This is achieved through a hop count discovery procedure inspired by the DSR algorithm to update the nodes’ hop-count. These multiple routes make it easy for the protocol to modify the chosen route from one path to another through electing the next relay nodes from a different path if this relay node is the best choice in the next hop forwarding set. Thus, this procedure avoids starting a new hop count discovery process. Initiating a hop count discovery happens only when all routes to all sinks fail. Our proposal uses the route information to update relay node information and guarantees that nodes responding to the P_holder_ have a path to one of the sinks to bypass the void nodes. 

The operation of the EEDOR-VA protocol is illustrated in Figure 2. When a source node has a packet to send, it first sends HCREQ and all its neighbors n1, n2, n3 and n4 receive it. Each of these neighbors rebroadcasts the request to its neighbors. When sinks s2 and s3 receive the request message, each sink generates an HCREP and sends it downwards. Consider the reply from s2. s2’s neighbors, n8 and n16, update their hop count and resend the HCREP to their neighbors. Each neighbor will update the HCREP with its depth and hop count and resend the reply hop by hop until the source node gets the reply. More discussion about hop count discovery is included in the next subsection. It is important to mention that the size and content of both HCREQ and HCREP in EEDOR-VA are reduced from those used in DSR to save energy and to allow multiple routes to the sink(s).

The EEDOR-VA protocol is divided into rounds; each round consists of three phases as given: a hop count discovery phase, a forwarding set formation phase and a data packet forwarding phase.

#### 3.3.1. Hop Count Discovery Phase

Hop count discovery is used to help any sensor node in the network to discover its hop count to sinks in the network, whether directly reachable within the transmission range or reachable via one or more hops through relay nodes. The general objective behind the hop count discovery phase is to obtain the hop count of all connected nodes in the network.

The hop count discovery phase consists of two procedures and assigns a hop count to the sensor nodes. Appendix A presents Algorithm A1, which illustrates both procedures of the hop count discovery. First, in the hop-count request procedure (lines 1–15), a source node generates the HCREQ consisting of a sequence number and the source’s ID and broadcasts it to its neighbors. Each neighbor node receives the HCREQ, updates its neighboring table with source ID (line 6) and maintains the request sequence number. If the request with this sequence number is received for the first time (line 7), then the neighbor node replaces the source’s ID with its ID in the HCREQ and rebroadcasts it to its neighbors (lines 8–9). Otherwise, the node just ignores the HCREQ (line 11). This procedure (lines 3–15) is repeated until the HCREQ reaches the destination (one of the sinks). 

Once a sink receives an HCREQ message, it will start a hop-count reply procedure that finds each receiving node’s hop count to the nearest sink. The sink hop count is initialized as 0 (line 16). The sink generates an HCREP consisting of sink’s ID, sink’s depth, sink’s hop count and the sequence number and broadcasts the reply (lines 17–19). When a node receives the HCREP and has previously received an HCREQ with this sequence number, the node then obtains the HCREP sender hop count from the reply message and compares it with its hop count (lines 21–23). If the node’s hop count is greater than hop count in the HCREP + 1, then this node will update its hop count by increasing the hop count in the received HCREP by 1 and assign it as its own hop count (line 24). 

After updating the HCREP receiver information, if this node is not the source node then the node updates the HCREP with its own ID, depth and hop count, and then rebroadcasts it to its neighbors (lines 25–27). If it is the source, the node then prepares for the next phase and will not rebroadcast the HCREP (lines 28–30). A sensor node will also ignore the HCREP if its hop count is less than or equal to the hop count attached to the HCREP (lines 31–33). The hop-count reply procedure (lines 20–35) is repeated until all nodes that have previously received the HCREQ receive the HCREP and rebroadcast it. The HCREP will eventually reach the source node. 

**Figure 4:**
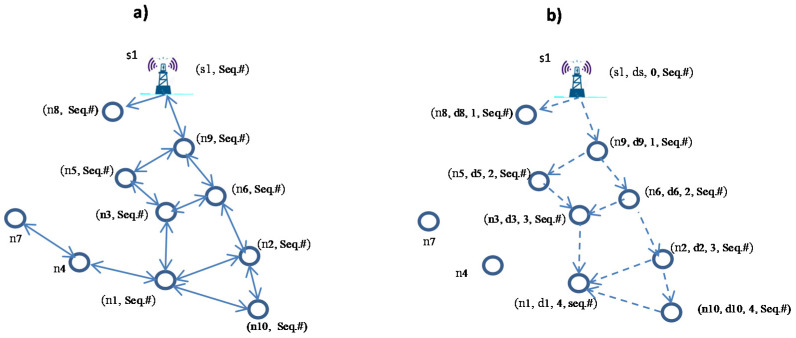
Energy Efficient Depth-based Opportunistic Routing with Void Avoidance for UWSNs (EEDOR-VA) hop count discovery paths. (**a**) Shows Hop Count Request (HCREQ) path from source node n1 to destination s1. (**b**) Shows Hop Count Reply (HCREP) path from destination s1 to source n1.

The following example in Figure 4 illustrates the hop count discovery phase of EEDOR-VA. In each round of our protocol, the nodes start collecting data from the surrounding environment. Whenever a node (n1) has a packet to transmit, the node will start hop count discovery to reach at least one of the sinks on the surface (s1). The current source (n1) broadcasts an HCREQ consisting of its ID (i.e., n1) and a sequence number to its one-hop neighbors (n2, n3, n4 and n10). Each neighbor node (n2, n3, n4) receives the HCREQ message, replaces its ID in the request and rebroadcasts it to its neighbors to continue the hop count discovery algorithm until sink (s1) receives the HCREQ message.

The solid arrows in Figure 4a represent the HCREQ path. Upon receiving the HCREQ, sink (s1) generates an HCREP (i.e., HCREP contains HCREP sender’s (ID, depth, hop count) and a sequence number) and broadcasts it to its neighbors (n8, n9). Node n8 ignores the reply message since it did not receive any HCREQ, while n9 updates its hop count and stores its information into the HCREP (i.e., nodes store their ID, depth and hop count) in addition to the sequence number. This is shown in Figure 4b as a combination next to each node. In this step, for example, node n9 has its ID n9 and it is at depth d9 with hop count 1; it will store (n9, d9, 1) and rebroadcast the reply to its neighbors. In each step, every neighbor node that receives a reply updates its hop count from the surface sink(s) if its hop count is greater than 1+ hop count in the received HCREP. Figure 3b shows the routes from sink s1 to node n1.

#### 3.3.2. Forwarding Set Formation Phase

The next step of our protocol is forming a forwarding candidate set that can forward the data packet to reach the sink(s) through the relay nodes. Algorithm A2 presented in Appendix B illustrates the forwarding set selection for any P_holder_ node (i.e., source or next-hop forwarding node). In the EEDOR-VA protocol, if the P_holder_ is not the sink (line 1), then we assume one of the two possible cases:

1. If one of the sinks is in a P_holder_ transmission range, then that sink will send HCREP with hop count 0 directly to the P_holder_ node (line 2). In this case, the P_holder_ ignores the forwarding set formation phase and forwards the data packet to the corresponding sink directly (lines 3–4).
2. If the current P_holder_ node cannot reach any of the sinks directly, then a group of relay nodes is selected to form a next-hop forwarding set (lines 5–10).

**Figure 5:**
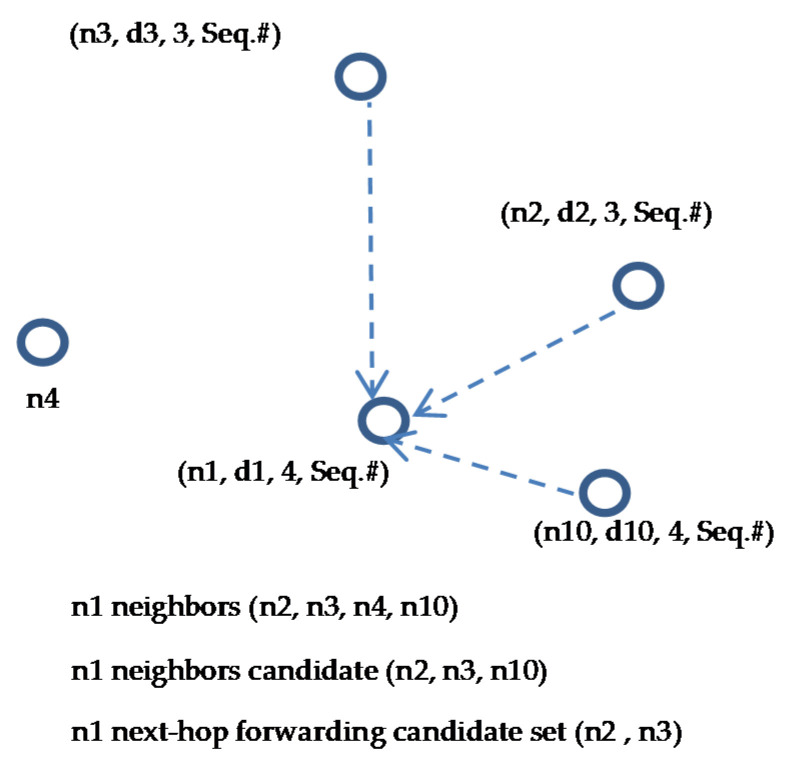
Forwarding set candidates selection.

A next-hop forwarder set is determined based on the extracted candidate information (IDs, depth and hop count) received with HCREP responses, as explained in the previous subsection. When the P_holder_ receives the HCREP from the candidate nodes, it checks the candidate’s hop count and compares it with its own hop count. The candidate node is added to the P_holder_‘s next-hop forwarding set only if its hop count is less than the P_holder_ hop count (lines 7–9), no matter if the candidate node has less depth than the P_holder_ or not. From the example presented in the previous subsection and illustrated in Figure 4, to determine node n1 next-hop forwarding set, node n1 checks the HCREP received from its neighbor nodes n2, n3 and n10. Node n1 compares its hop count (i.e., n1 hop count is 4 in the example) with its neighbors’ hop counts (i.e., n2 and n3 hop count is 3 while n10 hop count is 4) and includes the neighbors with lower hop counts in its next-hop forwarding set. Based on the forwarding set selection procedure, we can conclude that the forwarding set of node n1 consists of n2 and n3, as shown in Figure 5. Node n10 is excluded from the forwarding candidate set since its hop count is not less than n1′s hop count, while node n4 is excluded from the forwarding candidate set since it has no route to any of the sinks on the surface.

This technique leads to lower energy waste by removing the candidates with higher hop count from the forwarding set. Furthermore, the technique reduces the number of retransmissions as well as energy consumption. The P_holder_ node now knows its forwarding set nodes.

The current P_holder_ node then sorts its own forwarding set nodes in a list based on their hop count from the sink, and if two or more nodes have the same hop count from the sink, their depths will be used to break the tie (line 11). At each hop, only the list of sorted forwarding candidate IDs will be sent out along with the data packet. 

#### 3.3.3. Data Packet Forwarding Phase

In greedy protocols, the most appropriate node based on some criteria with the shortest holding time will transmit the data packet first. This approach can suppress other suitable candidates based on other criteria (e.g., energy, degree, hop count) from forwarding the data packets. As a result, the packet might become lost, decreasing the packet delivery ratio, and energy consumption may increase due to retransmission. Our proposal overcomes this weakness by checking if the next forwarding candidate node has a path to the sink(s) so it can carry on delivering the packet hop by hop or not. In this way, our proposal avoids selecting void/trapped nodes, which do not have a path to any of the sinks, to become one of the forwarding set candidates, as discussed in the previous step.

Once the neighbor nodes receive the data packet integrated with the sorted list of forwarding candidate IDs, each neighbor node checks if its ID is one of the IDs attached to the data packet. If the neighbor node cannot find its ID, then it simply drops the packet. Otherwise, the node has been chosen as a forwarding candidate and it starts the next step by computing its holding time, as explained below using Equation (9). In EEDOR-VA, the node’s hop count is considered as the first metric to determine the most appropriate forwarding node, and then the node’s depth will be used as a second metric in case of a tie. The most appropriate forwarding node will have a short holding time before transmitting the data packet to continue the forwarding procedure. If the most appropriate node successfully forwards the packet, and other forwarding candidates overhear the transmission, they will drop the packet. If not, the next node in the sorted list will transmit the data packet, and so on. These steps will be repeated until the data packet reaches the sink or all the candidate nodes in the forwarding set fail.

Holding Time calculation: We notice that a number of nodes may have the same depth and/or distance from the sinks or the sender node. This number increases with node density. A number of greedy protocols use the node’s depth to calculate the node’s holding time. Hence, the number of nodes with nearly equal transmission time increases and collisions and re-transmission also increase, resulting in excessive network energy consumption. Therefore, the holding time in our protocol, which is used to calculate the forwarding time, must fulfill the two following conditions: (1) A node’s holding time should decrease with a decrease in node hop count to the sink and node depth; (2) The holding time must also be sufficiently long to allow the lower priority candidate nodes in the forwarding set to hear the packet transmission by higher priority nodes before they forward the same data packet. 

Our proposed protocol satisfies the above-mentioned conditions. First, all the candidates in the forwarding set are sorted by the source in ascending order based on their hop count to the sink, and their depth is used to break any ties. Then, they are given a rank based on their place in the sorted list. A node’s rank increases with the increase in its hop count. Equation (9) is used to calculate the candidate’s node holding time (*HT*).

(9) $$HT=\frac{\left(2\times \frac{R_{tx}}{s}\right)}{DD}\times \left(R_{tx}-DD\right)\times \left(Rank-1\right)$$

where *R_tx_* is the node’s transmission range, *s* is the propagation speed of sound underwater (1500 m/s) and *DD* is the difference between the packet sender depth and the depth of its next forwarder.

In Equation (9), the first term aims to balance the propagation delays from the current P_holder_ node to all the candidates in the forwarding set. The second term of the equation is used to guarantee that the closer the candidate node is to the surface, the shorter the holding time. Finally, the third term assures a unique holding time for each candidate node based on its Rank value. Rank is the index of the node’s ID ordered based on their hop count as a first metric and depth as a second one to break the tie when two or more candidates have the same hop count and depth.

The candidate node with the smallest hop count will be on the top of the forwarding set list and have a rank (*Rank*) of 1. This node will start its transmission immediately because its holding time will be 0, while the other candidates will wait for a different period of time before transmitting the packet. Using Rank guarantees that all forwarding set nodes, including those with equal hop counts and depths, have different holding times so that their transmissions will not collide. Using node depth difference, DD, gives a larger holding time to nodes closer to the source, making short hops less likely.

As in EEDOR, the most appropriate node starts its transmission while the other nodes will suppress their transmission while their holding time is not expired. They will drop the packet if they hear the best forwarder node transmission. 

## 4. Simulation Results

In this section, we evaluate and analyze the performance of the EEDOR-VA protocol for UWSNs and compare it with the original EEDOR and DBR protocols through simulation experiments. All results were performed via simulations conducted in MATLAB. We aimed to enhance the reliability of the network through increasing the packet delivery ratio, which is achieved through our novel forwarding set selection by bypassing the void and trapped nodes in the packet forwarding procedure. In addition, we kept the network connectivity while reducing node energy consumption by minimizing the packet retransmissions, which decreases the packet collisions. In this work, two different scenarios were simulated using the network topologies that exist in [15,19]. These two topologies can be classified as networks with a small number of nodes. Since a void area is more likely to exist in networks with a small number of nodes, we choose to evaluate the EEDOR-VA with this type of network. In fact, since EEDOR-VA works well with these network topologies, it is pertinent to state that it also works well when increasing the number of nodes due to the low probability of void areas.

The metrics used for performance evaluation are elaborated as follows:

1. Total Energy Consumption (*E_total_*) denotes the total energy consumed in hop count discovery HCREQ and HCREP messages and data packets delivery, including the transmitting, receiving, and idling energy consumption of all nodes in the network. The total energy consumption is a cumulative summation that starts at 0. This can be calculated mathematically as:
  (10) $$E_{total}=\overset{rounds}{\underset{i=1}{\sum }}\overset{n}{\underset{j=1}{\sum }}\left(E_{init}-E_{resd}\right)$$
  where *rounds* is the number of simulation rounds, *n* is the number of underwater sensor nodes, *E_init_* is the sensor node initial energy, and *E_resd_* is the sensor node residual energy.
2. Mean Energy consumption per node (*E_Mean_*) is defined as the average of the total energy consumption. Mathematically, *E_Mean_* is presented as:
  (11) $$E_{Mean}=E_{total}/n$$
  where *E_total_* is the total energy consumption calculated by Equation (10) and *n* is the number of deployed nodes.
3. Total number of transmissions (*N_trans_*) denotes the total number of nodes that forward the data packet starting from the source node to reach one of the sinks on the surface. *N_trans_* can be presented mathematically as follows:
  (12) $$N_{trans}=\overset{rounds}{\underset{i=0}{\sum }}FN$$
  where *rounds* is the number of simulation rounds and *FN* is the number of transmitting nodes in one round.
4. Packet Delivery Ratio (*PDR*) is defined as the ratio of the total number of distinctive packets received successfully at any of the sinks (*P_success_*) to the total number of generated packets (*P_sent_*). We calculate *PDR* mathematically as:
  (13) $$PDR=P_{success}/P_{sent}$$

### 4.1. Scenario 1:

#### 4.1.1. Simulation Parameters

In this subsection, we summarize the general parameters we used in our simulation experiments. In the first scenario, the simulation parameters were initialized as in [15]. All nodes are homogeneous in terms of initial energy and transmission range. The power consumed by nodes is 2 W, 0.1 W and 0.01 mW in transmitting, receiving and idling, respectively; they are similar to those on a commercial acoustic modem, LinkQuest UWM1000 [34]. For DBR, we used a depth threshold of zero, as in [20]. Other simulation parameters are summarized in Table 2 below. In this scenario, the void avoidance protocol EEDOR-VA is compared with EEDOR proposed in our previous work [15], where EEDOR was shown to be superior to various other algorithms. We also consider the well-known DBR in our comparison results because it is the first depth-based protocol. Statistical and comparison results between the three protocols presented in this section were obtained using 100 runs of our simulation.

#### 4.1.2. Results and Analysis

**Figure 6:**
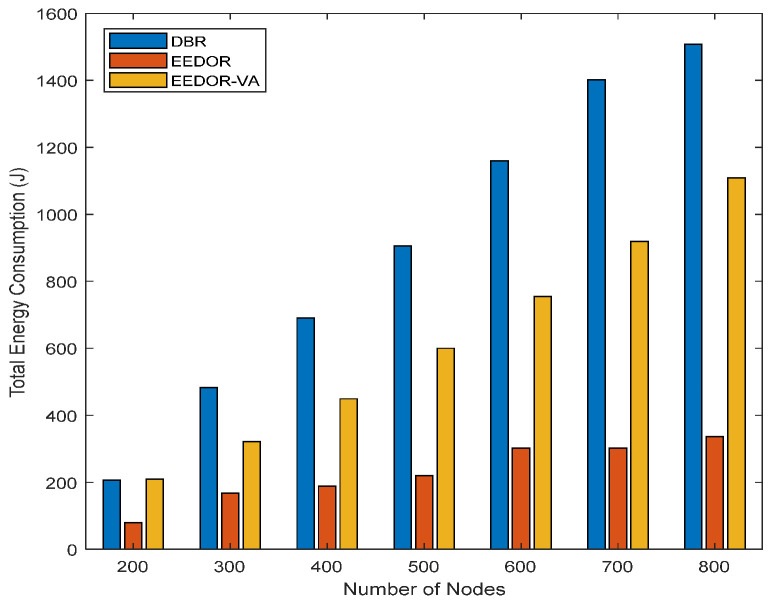
Total Energy Consumption vs. Number of Nodes.

**Total Energy consumption:**Figure 6 illustrates the total energy consumption for the three protocols. It can be observed that DBR has the largest total energy consumption since it depends on the greedy flooding technique to forward the data packets from the source nodes to reach the surface sink(s). This greedy technique used by DBR results in a number of transmissions occurring at the same time, which makes collisions between the transmitted packets more likely. Moreover, the depth threshold mechanism used by DBR for selecting next-hop forwarding nodes affects the overall energy consumption by reducing the number of relay nodes. Reducing the number of relay nodes increases the probability of packet loss and therefore increases retransmissions, which increases the energy consumption. In contrast, the total energy consumed by EEDOR is almost constant because the network density has an insignificant effect on our next-hop forwarding method. On the other hand, EEDOR-VA’s total energy consumption also increases with increasing the density of the network. The difference between the total energy consumption in the three protocols, especially between DBR and the other two, EEDOR and EEDOR-VA, increases rapidly with the increase in the density of the nodes. The large variation in energy consumption shown in Figure 6 among the three protocols as the network density increases is caused mainly by an increase in the size of the forwarding set and redundant transmissions in DBR as the network density increases. EEDOR and EEDOR-VA constrain the number of redundant transmissions due to the coordination method based on the proposed holding time. However, the hop count discovery procedure in EEDOR-VA gives the protocol an extra energy cost. 

**Figure 7:**
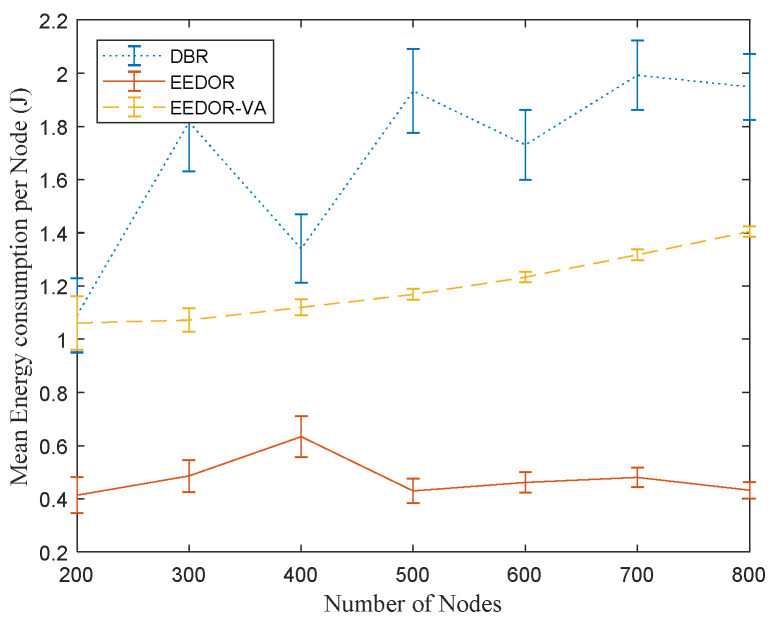
Mean Energy Consumption per Node vs. Number of Nodes.

The 95% confidence interval error bars in Figure 7 also show that our EEDOR-VA protocol has a smaller error range in mean energy consumption per node than the other two protocols. Additionally, the EEDOR-VA error range decreases as the topology density increases. The small error range indicates that the energy consumption is more balanced between all nodes. The DBR large variation in mean energy consumption per node as shown by the error bars in Figure 7 happens because some of the nodes, especially near the surface, retransmit more often than others because DBR does not incorporate any energy balancing strategies. EEDOR and EEDOR-VA waiting time techniques in the forwarding set selection phase help in choosing the next-hop forwarder and balancing the energy consumption by suppressing the retransmissions.

**Figure 8:**
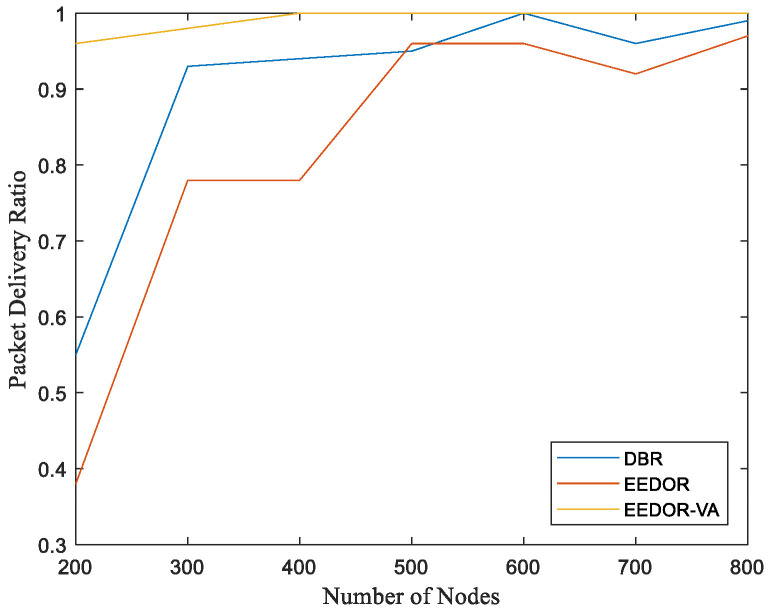
Packet Delivery Ratio (PDR) vs. Number of Nodes.

**Packet Delivery Ratio (PDR):** In this network topology, a high value of packet delivery ratio of routing protocols means that the network is unlikely to have void areas. The PDR is high because increasing the number of nodes increases the number of forwarding nodes in the routing path and, as a result, increases the PDR. We can observe from Figure 8 that the packet delivery ratio of EEDOR-VA is always higher than that of other routing protocols, mainly because it omits all the routes that lead to a void area. Our technique can deal with the void problem without implementing any recovery mode, which eliminates the recovery mode high overhead.

Furthermore, the packet delivery ratio of DBR and EEDOR is not as high as EEDOR-VA because both the DBR and EEDOR protocols do not consider if there is at least one route between the source and the sink(s) or not. If no route exists, packet forwarding failure is increased since at some point in data transmission, the current packet holder node cannot find any appropriate node with less depth than itself. We can also observe from Figure 8 that DBR has a better PDR than EEDOR because it uses a greedy mechanism to flood the network with data packets.

The redundant packets, which happen because the flooding mechanism and resulting multiple paths, increase the probability of successful packet delivery and, as a result, the PDR, while in EEDOR, the current node selects its forwarding set based on the neighboring node depths without identifying void/trapped nodes. If the next forwarder is a void/trapped node (and therefore cannot find any node with less depth than itself), that node drops the received packet, which decreases the EEDOR packet delivery ratio. Finally, we conclude that the higher PDR for EEDOR-VA comes at an extra expense of energy cost compared with EEDOR. However, it still achieves higher PDR using less energy consumption than DBR.

### 4.2. Scenario 2:

#### 4.2.1. Simulation Parameters

This scenario presents the simulation of the second network topology. The list of the configuration parameters used in our experiments is presented in Table 3. These simulation parameters were initialized as in [19]. Moreover, the power consumed by nodes is 0.8 W and 0.01 mW in receiving and idle modes, respectively. The depth threshold of DBR is one fourth of the maximum communication ranges. In our simulation, a source node was randomly selected among all the randomly deployed nodes. In each simulation run, the destination of all data packets is one of the 16 sinks randomly deployed on the water’s surface. The statistical and comparison results between EEDOR, EEDOR-VA and DBR presented in this section were obtained using 30 runs of our simulation.

#### 4.2.2. Results and Analysis

**Figure 9:**
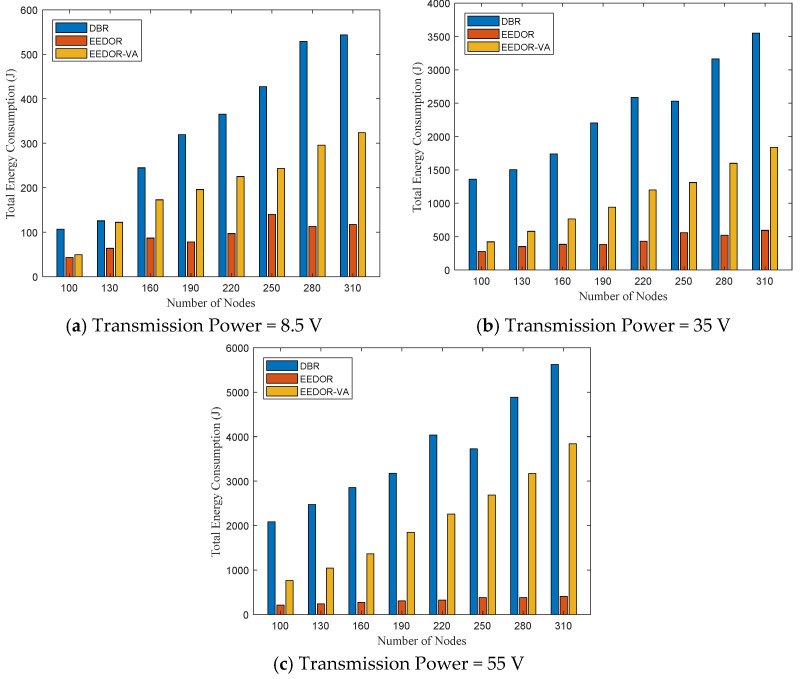
Total energy consumption comparison at different transmission powers.

**Total Energy consumption:** the total energy consumption of the three protocols is illustrated in Figure 9. As we can see in the three subfigures, the transmission power level and node density have a direct impact on the total energy consumption. That is, increasing either the power level or the node density increases the connectivity between the deployed nodes in the network topology. A higher connectivity means a larger number of nodes participating in the data forwarding procedure, thereby increasing the energy consumed. Moreover, we noted from the figure that, for example, for 100 nodes, the total energy consumption increased by approximately 10 times when we increased the power level from 8.5 W to 35 W, and about 17 times when increased from 8.5 W to 55 W, while, for example, in the topology with a transmission level of 8.5 W, increasing the density of the network from 100 nodes to 310 nodes increases the total energy consumption by only about 7 times. Consequently, the power level has more effect on the EEDOR-VA total energy consumption than the network density.

**Figure 10:**
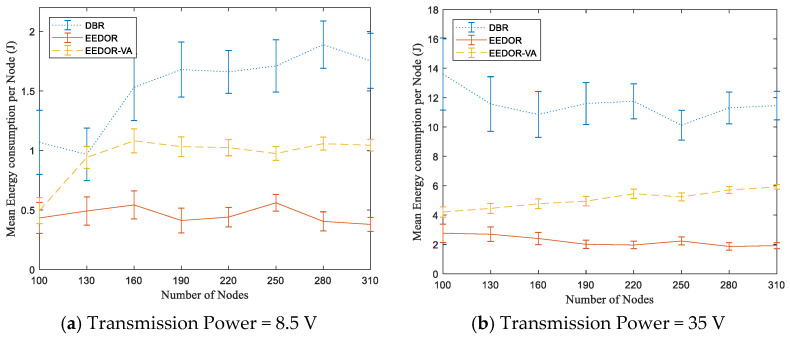

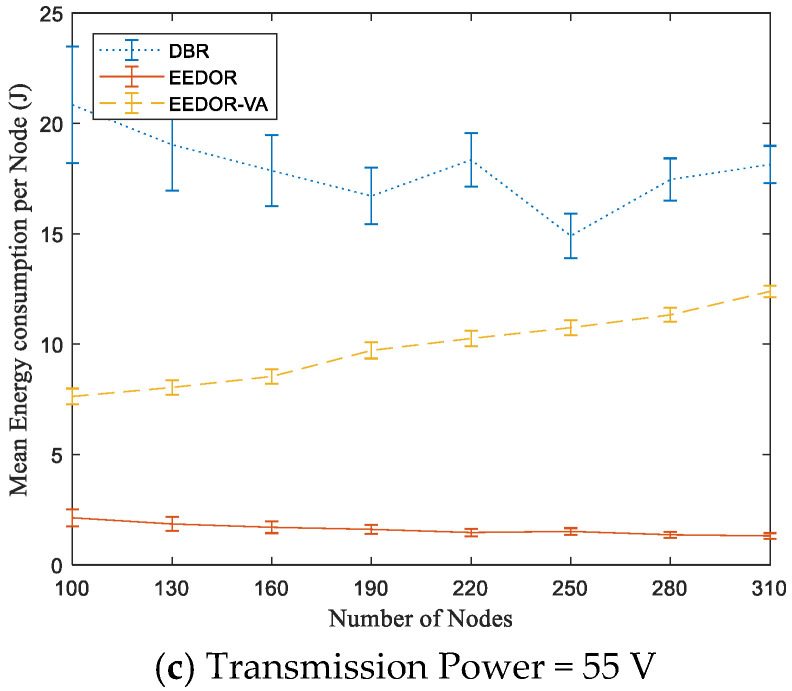
Comparison of mean total energy consumption per node at different transmission powers.

The 95% confidence interval error bars in Figure 10 show that our EEDOR-VA protocol has a smaller error range, mainly with a low transmission power level. Additionally, the EEDOR-VA error range decreases as the network density increases. The small error range indicates that the energy depletion is more stable between all the nodes in the topology. In DBR, the large variation in mean energy consumption per node occurs because of the flooding technique, where some of the nodes with lesser depth, especially those near the surface, retransmit more frequently since DBR does not incorporate any energy balancing strategies. EEDOR and EEDOR-VA waiting time techniques assist in the next-hop forwarders collaboration to transmit the data packet and balance the energy consumption by suppressing the retransmissions.

**Figure 11:**
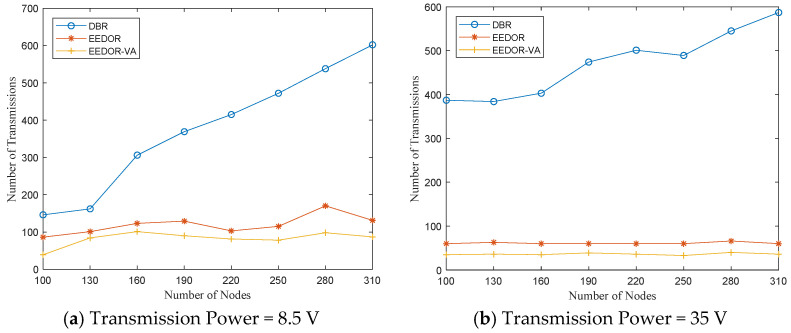

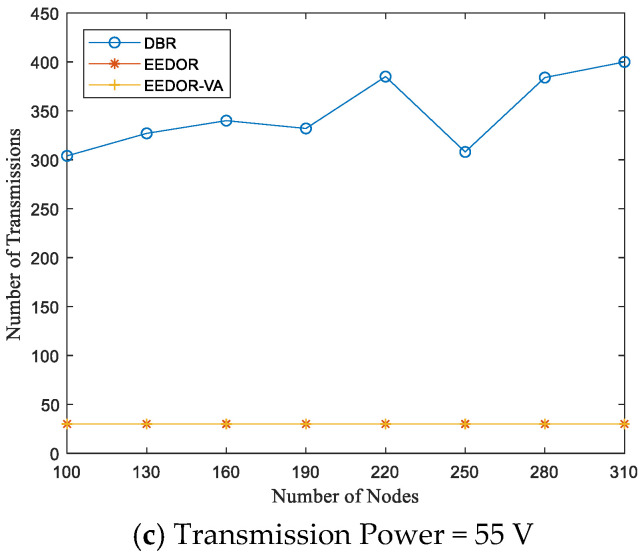
Number of transmissions comparison at different transmission powers.

**Total Number of Transmissions:** To clarify the extensive variance between the total consumed energy of DBR and both of our protocols, EEDOR and EEDOR-VA, we calculate the total number of nodes participating in data packet transmissions starting from the source node until reaching the sink for all three protocols using the three power levels. Figure 11a–c shows that DBR has the largest total number of nodes that transmit the packet, and this number increases with increasing the network density. The increase in the total number of transmitting nodes in the DBR protocol as the network density increases happens due to the greedy flooding technique used by the protocol to forward the data packet. 

In contrast, both EEDOR and EEDOR-VA use the proposed waiting time to allow the lower priority forwarding nodes to hear the higher priority node transmissions. This yields a much smaller total number of transmitting nodes. Moreover, the subfigures show that the numbers of transmitting nodes for EEDOR and EEDOR-VA become closer to each other with a higher transmission power level. We also conclude from Figure 11 that for EEDOR and EEDOR-VA, the total number of transmitting nodes is relatively constant with the network density, especially with high transmission power levels, while it increases greatly with network density for DBR.

**Figure 12:**
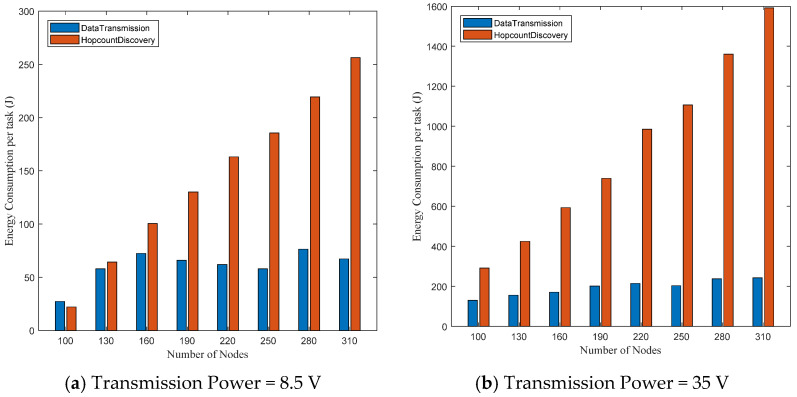

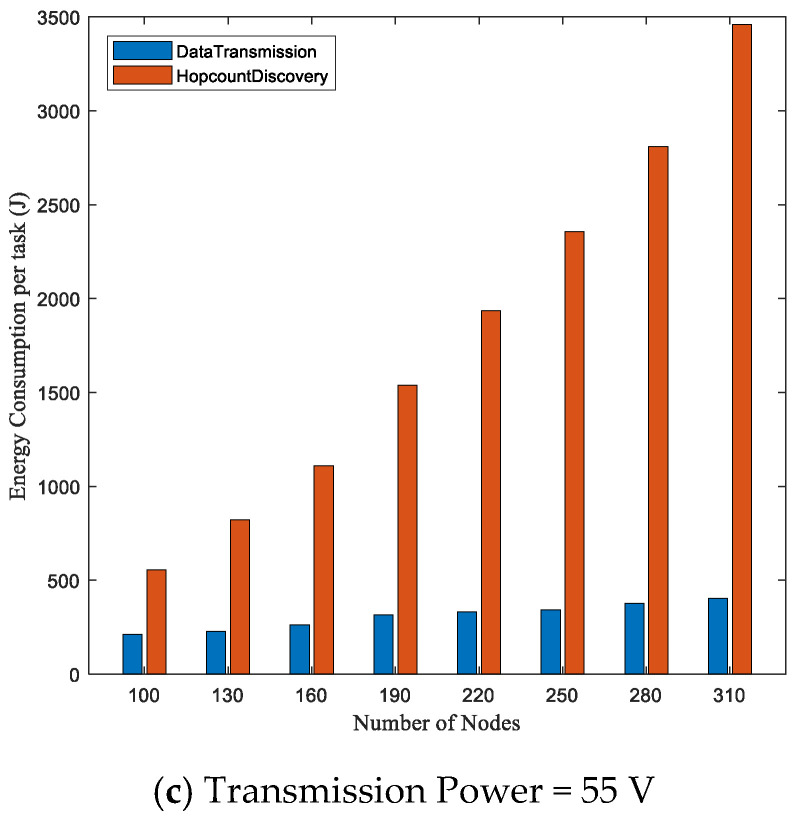
EEDOR-VA total energy consumption per task at different transmission power levels.

Figure 11 shows that the number of nodes that actually transmit the data packets using EEDOR-VA is less than the number of transmitting nodes when using EEDOR. These results motivated us to study the extra energy consumed by EEDOR-VA during both hop count discovery and data transmission. Figure 12 illustrates the total energy consumption for the hop count discovery and data transmission processes. As shown in Figure 12a, in the network topology with 100 nodes, the energy consumed in data transmission is a little more than that consumed in hop count discovery. This similarity in energy consumption happens because the HCREQ and HCREP do not broadcast through the whole network and their size is much smaller than the data packets. Relative energy consumption is reversed when the density of the nodes increases, which increases the number of the nodes that can communicate with each other. This increase in node connectivity means more nodes will rebroadcast HCREQs and HCREPs to cover all connected nodes, while the data packets will be transmitted hop-by-hop through only the relay nodes with discovered hop count to deliver them to the destination. The same explanation is also true when using higher transmitting power levels, as shown in Figure 12b,c. Thus, the EEDOR-VA hop count discovery process is responsible for most of the total energy consumption of the network.

**Figure 13:**
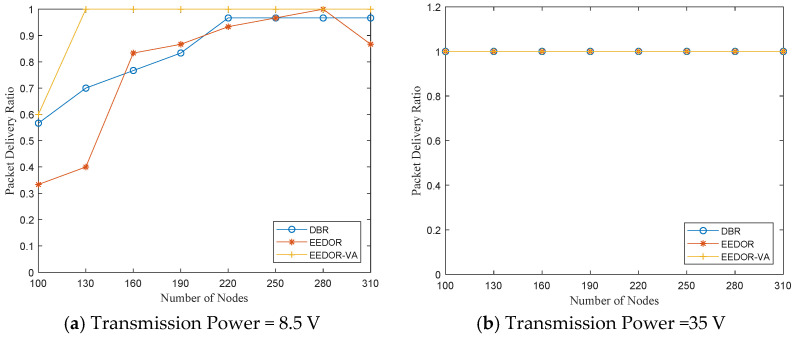

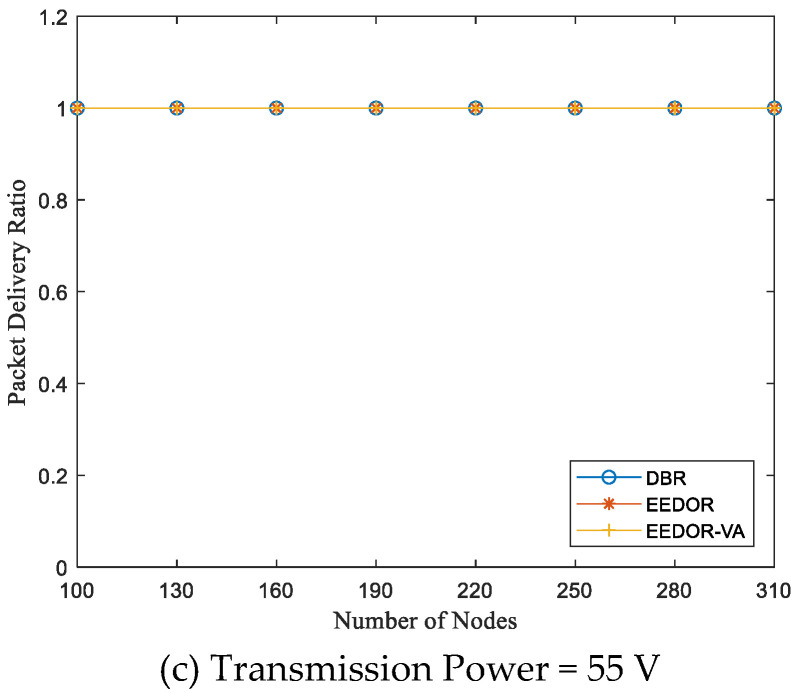
EEDOR-VA Packet Delivery Ratio (PDR) at different transmission powers.

**Packet Delivery Ratio (PDR):** In this network topology, a lower value of packet delivery ratio of routing protocols is expected since the nodes are deployed far apart from each other, which makes void areas in the network more likely. Moreover, since the sensor nodes may be out of the communication range of each other, the forwarding sets may contain a small number of candidates or they will be empty and therefore will affect the PDR. We assess the performance of the comparison protocols by decreasing the void nodes in the network through increasing the number of deployed nodes and/or increasing the transmission power level. We simulate the three protocols at three transmitting power levels. Simulation results are shown in Figure 13. First, Figure 13a shows that using the smallest power level and the smallest number of nodes in our experiment, EEDOR-VA outperforms DBR and EEDOR. Increasing the number of nodes at the same power level for EEDOR-VA in Figure 13a or raising the power level with a different number of nodes for the three comparison protocols will help to increase the PDR to the maximum, as shown in Figure 13b,c. This occurs because an increase in the number of nodes or an increase in the power level leads to an increase in the number of next-hop forwarding candidates, and this helps to increase the probability of delivering the packet successfully.

It is important to mention that, referring to [19], the power control method used by PCR protocol helps to increase the connectivity between the nodes. This increases the number of forwarding candidates. In DBR, the depth threshold mechanism and the large number of disconnected nodes tend to decrease the number of forwarding candidates. This decrease in forwarding candidates explains why, in [19], DBR outperformed the PCR protocol in energy consumption. It also explains the low PDR for DBR compared with the high PDR obtained by the PCR protocol. On the other hand, when we compare DBR with EEDOR-VA, the redundant packets and retransmissions in DBR are the reason for the extra amount of depleted energy. Additionally, the collisions in the DBR protocol are caused mainly by redundant packets, which increases the probability of packets being lost and decreases the PDR. For EEDOR-VA, the forwarding candidate priority and holding time reduces the total energy consumption through minimizing the redundant transmissions, and the hop count discovery process maximizes the PDR by guaranteeing that the packet is delivered successfully. It is important to note that the simulation results in both scenarios presented above show that our proposed EEDOR-VA outperforms DBR and PCR in terms of energy usage and PDR.

### 4.3. Impact of the Number of Sinks and Deployment on EEDOR-VA

We examine the effect of various network architecture designs with (1) single and multiple sinks and (2) different sink deployment techniques on EEDOR-VA performance.

**Figure 14:**
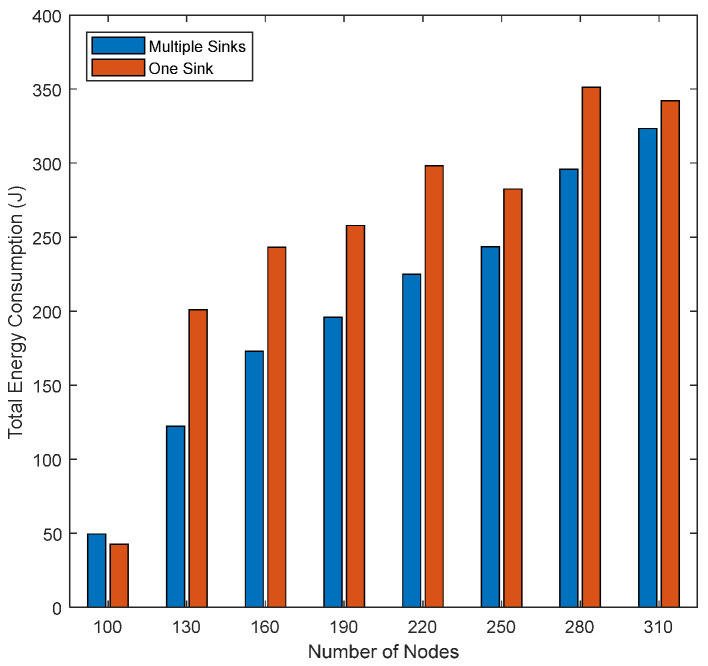
Impact of the number of sinks on the total energy consumption.

**Number of Sinks:** to examine the impact of the number of randomly deployed sinks on the network performance, scenario 2 with 16 sinks for the multi-sinks’ architecture and the transmission power level = 8.5 watt is used. Figure 14 shows the total energy consumption for single and multiple sinks. We see that the total energy consumption of single sink structure networks is higher than the total energy consumption of multi-sink structure networks. The reason is that in EEDOR-VA, the number of sinks impacts the hop count discovery and forwarding procedure. Using multiple sinks increases a node’s chance of reaching one of the sinks through fewer hops, which means decreasing the number of HCREQs required to reach one of the sinks. Moreover, data packets are transmitted via the nodes with discovered hop count to reach the closest sink. In contrast, in the one sink architecture, the HCREQ will be rebroadcasted until that single sink is reached or until rebroadcasted from all the nodes in the network. Consequently, more energy is consumed in single sink networks than multi-sink networks.

**Figure 15:**
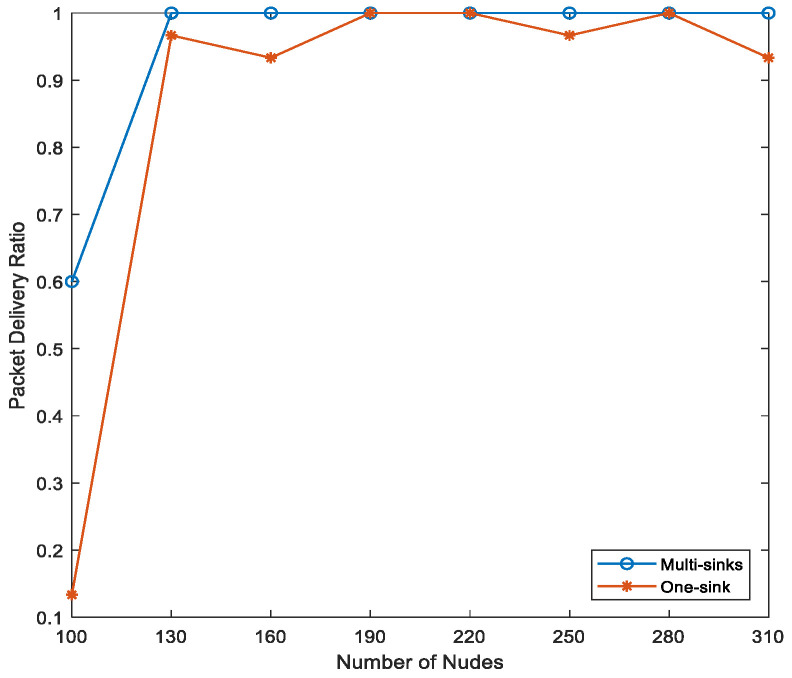
Impact of number of sinks on the packet delivery ratio.

Figure 15 shows that the packet delivery ratio for both single- and multi-sink architectures is quite variable in the network topologies with the lowest number of deployed nodes. According to the figure, when using EEDOR-VA with the lowest number of nodes topology (i.e., 100 nodes), increasing the number of deployed sinks on the sea surface will increase the number of nodes that can communicate with one of these sinks. Consequently, the chance of one of the sinks receiving the data packet will be increased, resulting in a higher packet delivery ratio in a multi-sink topology. In contrast, with the same number of nodes (i.e., 100 node topology) and one-sink network architecture, all data packets must reach that single sink to be successfully delivered. The number of nodes that cannot reach that single sink is high. This effect explains the very low packet delivery ratio shown in Figure 15 for a 100 node network. On the other hand, the number of sinks has an insignificant effect on the PDR in the topologies with a greater number of deployed nodes, and this is due to the increased probability of reaching the sink(s).

**Figure 16:**
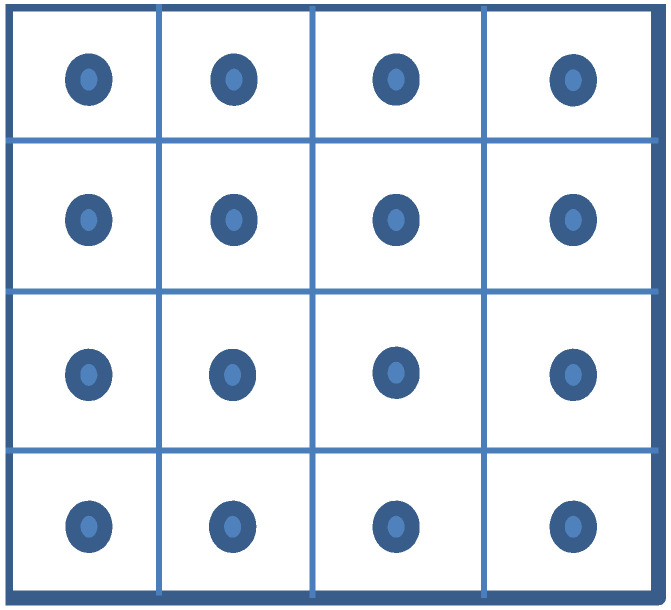
Pre-planned deployment sinks.

**Sinks deployment:** Two sink deployment methods, pre-planned and random, are used to examine the EEDOR-VA performance. Random deployment is widely used in the literature, while the pre-planned deployment is used in [19]. The pre-planned method used in our simulation is illustrated in Figure 16. In this method, all the sinks are deployed in specific locations with the same distance from each other to cover the area of interest on the sea surface, while in the random deployment, the sinks are distributed randomly on the sea surface. In this method, the distance between any two sinks is variable. 

**Figure 17:**
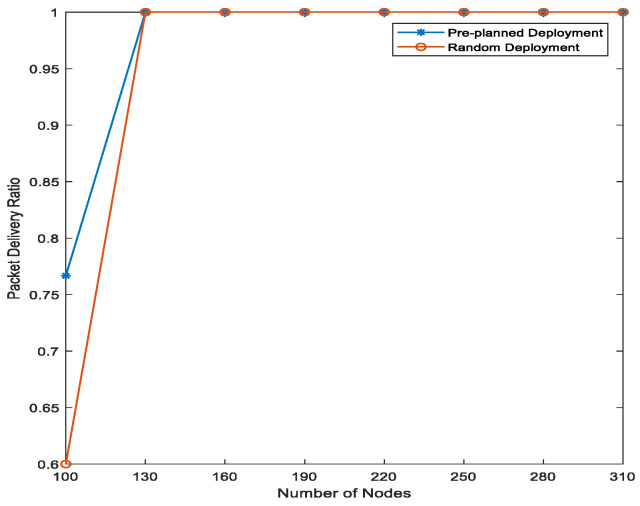
Impact of sink deployment on the EEDOR-VA packet delivery ratio.

Figure 17, which presents the simulation results related to the PDR for both sink deployment methods, shows that the deployment method has no noticeable impact on our proposed protocol EEDOR-VA. The reason behind this is that as long as there is a path between the forwarder node and any of the deployed sinks, the data packet will be transmitted. In other words, the sink deployment methods have nothing to do with the data packet forwarding process we implemented in the EEDOR-VA protocol.

**Figure 18:**
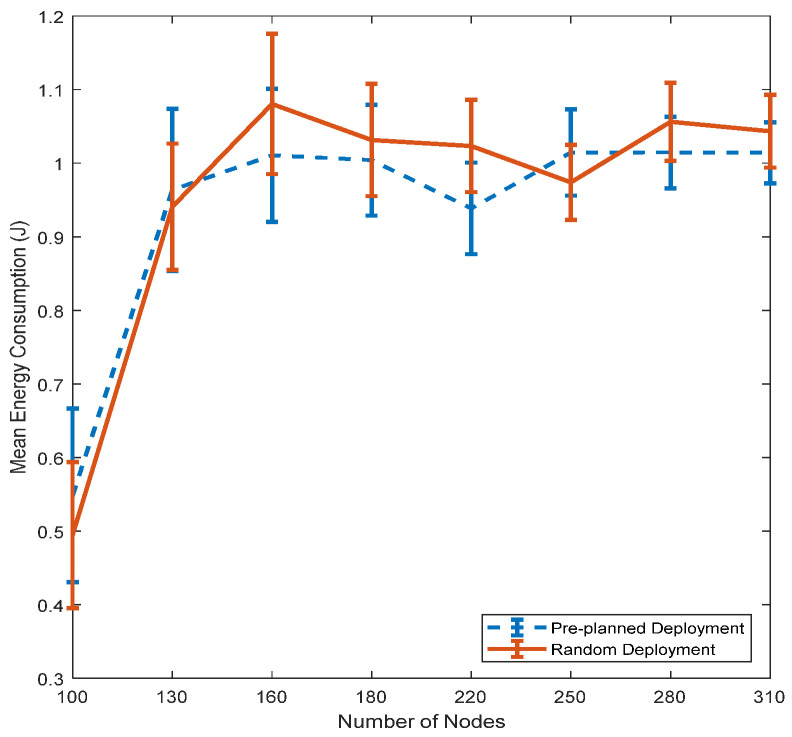
Impact of sink deployment on the EEDOR-VA energy consumption.

The comparison of energy consumption between both deployment methods is illustrated in Figure 18. As shown in the figure, the 95% confidence interval error bars show that there is no significant difference in the energy consumption between the two deployment methods. The energy consumption is similar because the HCREQ and HCREP messages in the hop count discovery phase, which are responsible for the largest amount of the total consumed energy, are broadcasted between the random distributed sensor nodes in the network topology. In other words, the energy consumed in the hop count phase is related to the sensor nodes and has little to do with the sinks. Moreover, the data forwarding process in EEDOR-VA is not affected by the sink deployment method since the relay nodes that are used for forwarding the data packets are defined during the hop count discovery phase.

## 5. Conclusions

In this paper, we propose EEDOR-VA, a void avoidance protocol. EEDOR-VA increases the packet delivery ratio relative to our previous protocol EEDOR [15] and the established DBR [20], especially in networks with a small number of nodes where the void areas are most likely to occur, as we presented in Section 4. In addition, EEDOR-VA improves the reliability of the network by detecting void/trapped nodes in advance of data transmission using the hop count discovery process. It also reduces the number of nodes that actually transmit the data packets by using *Rank* to distinguish between node holding times. This approach helps to decrease the energy expenditure in the data transmission process as well as packet collision and its associated cost.

The EEDOR protocol presented in [15] is localization free, as is its enhanced version EEDOR-VA. EEDOR was the superior protocol when compared to various other depth-based algorithms in [15]. In this work, we examined EEDOR-VA in two dissimilar network topologies in addition to different transmission power levels in one of the simulated topologies. 

The analyses of experimental simulation results show that EEDOR-VA enhances network performance in terms of energy consumption, packet delivery ratio and the number of nodes that actually complete the transmitting process. We further analyzed the impact of single and multiple sinks as well as two different sink deployment techniques on the EEDOR-VA performance in terms of total energy consumption and PDR.

## Figures and Tables

**Table 1:** Summary comparison of different existing protocols.

Protocol	Advantage	Disadvantage
VAPR [16]	Reduces end-to-end delay, incorporates void handling technique.	Has high energy consumption, periodic beacons cause network overhead.
GEDAR [17]	Uses a network topology control method to handle voids, increases the connectivity of the network, reduces the number of packet retransmissions.	Has high energy consumption due to depth adjustment technique, requires frequent topology changes; exhausts node energy in physical movement, decreasing network lifetime.
VHGOR [18]	Handles void nodes in two ways (i) Convex void handling and (ii) Concave void handling (or) recovery mode.	Consumes restricted resources due to beacon exchange, hidden terminal problem and duplicate packets.
CPR [19]	Improves the link quality at each hop, reduces the number of packet transmissions in dense networks, and increases the packet delivery ratio.	Consumes significant energy in the forwarding set selection phase through a power control mechanism, has high communication overhead due to broadcasting the beacon messages with different power levels.
DBR [20]	Has a high packet delivery ratio, decreases the number of forwarding set candidates using a depth threshold technique.	Has high energy consumption overall due to flooding technique, redundant packets and collisions. Does not handle voids.
HydroCast [21]	Reduces end-to-end delay. Has a high packet delivery ratio. Uses recovery path to handle voids.	Has high energy consumption due to the detour path process, and high overhead because it requires two hop neighboring node information.
IVAR [22]	Eliminates all the routes leading to a void area without needing to switch to recovery mode.	Suffers from the hidden node problem, has high energy consumption due to redundant packet transmissions.
WDFAD-DBR [23]	Decreases energy consumption, handles packet duplication and expected next hop depth, reduces packet sticking in void holes.	Suffers from void area problem due to trapped nodes, has high communication overhead caused by control packet exchange and packet retransmission.
EVA-DBR [24]	Addresses hidden node problem in some cases, balances energy consumption and latency, detects void and trapped nodes.	Consumes node resources due to neighbor’s information exchange, duplicates packet transmissions in spares networks, and suffers from the hidden node problem in some cases.
EDOVE [25]	Considers energy level as one of its parameters, reduces energy consumption and avoids energy holes.	Has high communication overhead due to information exchanges, duplicates packet transmissions, has high energy consumption and does not handle void areas completely.
EEDOR [15]	Extends network lifetime using an energy efficient protocol, decreases the redundant packet transmissions and collisions, and reduces the forwarding set size by excluding nodes with greater depth.	Does not handle void areas.

**Table 2:** Summary of Scenario One Simulation Parameters.

Parameters	Value
Network size	500 m × 500 m × 500 m
Number of sinks	5
Number of nodes	200–800
Maximum transmission range	100 m
Initial energy	70 J
Data packet size	50 bytes
Data rate	104 bps
Frequency	25 kHz

**Table 3:** Summary of Scenario Two Simulation Parameters.

Parameters	Value
Network size	3000 m × 1500 m × 3000 m
Number of sinks	16
Number of nodes	100–310
Distribution	Random
Initial Energy	70 J
Transmission powers	(8.5, 35, 55) W
Maximum transmission range	(500, 1200, 2000) m
Data packet size	150 bytes
Frequency	37.400 kHz
Data rate	18,700 bps
s (shipping)	0.5
w (wind)	4

## Data Availability

Not applicable.

## Appendix A

**Algorithm A1** Updating Node Information
**Hop-count request procedure:**
1: node (n_i_) has a packet to transmit
2: n_i_ generates HCREQ message //Node n_i_ HCREQ (IDn_i_, sequence#)
3: n_i_ broadcasts HCREQ
4: node n_j_ receives the HCREQ
5: *IF* n_j_ is not a sink
6: n_j_ updates its neighboring table
7: *IF* n_j_ received the HCREQ for the first time
8: n_j_ replaces its ID in the HCREQ //Node n_j_ HCREQ (IDn_j_, sequence#)
9: n_j_ rebroadcasts the HCREQ
10: *ELSE*
11: n_j_ Ignores the HCREQ
12: *ENDIF*
13: *ELSE*
14: call Hop-count reply procedure
15: ENDIF
**Hop-count reply procedure:**
16: sinks hop count = 0
17: sink receives HCREQ
18: sink initiates HCREP // HCREP format (sink’s ID, sink’s depth, sink’s hop-count, sequence#)
19: sink broadcasts the HCREP
20: node n_i_ receivers the HCREP
21: *IF* n_i_ has received the associated HCREQ previously
22: n_i_ extract the hop count from HCREP
23: *IF* n_i_’s hop count > extracted hop count+1
24: n_i_’s hop count = the extracted hop count + 1
25: *IF* n_i_ is not the source node *THEN*
26: n_i_ updates the HCREP (n_i_’s ID, n_i_’s depth, n_i_’s hop count, sequence#)
27: n_i_ rebroadcasts the HCREP
28: *ELSE*
29: prepare for the forwarding set selection procedure
30: *ENDIF*
31: *ELSE*
32: n_i_ Ignores the HCREP
33: *ENDIF*
34: *ELSE*
35: n_i_ Ignores the HCREP
36: *ENDIF*

## Appendix B

**Algorithm A2** Forwardingsetselection
1: source receives HCREP
2: *IF* source receives HCREP directly from the sink // sink in the source transmission range
3: source transmit the packet // No forwarding set formation needed
4: Packet delivered to the sink
5: *ELSE* // source receives HCREP via relay nodes
6: *FOR* n_i_ ∈ source neighbors
7: *IF* ni hop count < source hop count
8: add n_i_ to source forwarding set
9: *ENDIF*
10: *ENDFOR*
11: Sort source Forwarding set // Hop count is considered first then depth in case of a tie
